# Bio-Inorganic Studies on the Fe(II) Sparfloxacin Complex

**DOI:** 10.1155/MBD.2002.1

**Published:** 2002

**Authors:** Swati Jain, N. K. Jain, K. S. Pitre

**Affiliations:** 1 Department of Chemistry Dr. Harisingh Gour University Sagar MP 470 003 India; 2 Department of Pharmaceutical Science Dr. Harisingh Gour University Sagar MP 470 003 India

## Abstract

The qualitative and quantitative analysis of an antibiotic drug, 5-amino-1 cyclopropyl-7 (cis-3, 5 dimethyl-1-piperazyl)-6,8- dihydro-1, 4 dihydro-4-oxo-3-quinoline carboxylic acid (Sparfloxacin, SFX) and its pharmaceutical formulation i.e.sparx-100 tablet, has been done using polarographic and amperometric methods. Complexation behavior of SFX with Fe(II), both in solid and liquid phases has been studied by elemental analysis, IR.-spectra and polarographic and amperometric methods. SFX produces a single cathodic reduction wave in 0.1 M ammonium tartrate (supporting electrolyte) at pH 6.0 ±0.1. The wave is diffusion controlled and wave height is proportional to the concentration of SFX. The complex is also reversibly reduced at the electrode surface with diffusion-controlled kinetics. The stoichiometry of the Fe(II)- SFX complex is 1:1. Antibacterial studies on the drug and its metal complex have been performed against different bacteria. The observed results revealed the complex to be more potent in its antibacterial activity as compared to the parent drug. On the basis of observed results it could be concluded that the prepared Fe(II)- SFX complex may be recommended to the therapeutic experts for its possible use as a more potent antibiotic drug.


### Bio-Inorganic Studies on the Fe(II) Sparfloxacin Complex

Swati Jain*, N.K.Jain** and K.S.Pitre*

**Department of Chemistry,*
***Department of Pharmaceutical Science,*
*Dr. Harisingh Gour University,*
*Sagar (MP) 470 003, India.*

**ABSTRACT**

The qualitative and quantitative analysis of an antibiotic drug, 5-amino-1 cyclopropyl-7 (cis-3, 5 dimethyl-1-piperazyl)-6,8- dihydro-1, 4 dihydro-4-oxo-3-quinoline carboxylic acid (Sparfloxacin, SFX) and its pharmaceutical formulation i.e.sparx-100 tablet, has been done using polarographic and amperometric methods. Complexation behavior of SFX with Fe(II), both in solid and liquid phases has been studied by elemental analysis, IR.-spectra and polarographic and amperometric methods. SFX produces a single cathodic reduction wave in 0.1 M ammonium tartrate (supporting electrolyte) at pH 6.0 ±0.1. The wave is diffusion controlled and wave height is proportional to the concentration of SFX. The complex is also reversibly reduced at the electrode surface with diffusion-controlled kinetics. The stoichiometry of the Fe(II)-SFX complex is 1:1. Antibacterial studies on the drug and its metal complex have been performed against different bacteria. The observed results revealed the complex to be more potent in its antibacterial activity as compared to the parent drug. On the basis of observed results it could be concluded that the prepared Fe(II)-SFX complex may be recommended to the therapeutic experts for its possible use as a more potent antibiotic drug.

**Key words**: Biometal, Sparfloxacin, Polarography.

### INTRODUCTION

SFX, 5-amino-1cyclopropyl-7 (cis-3,5 dimethyl-1-piperazyl)-6,8- dihydro-1, 4 dihydro-4-oxo-3-quinoline carboxylic acid (an antibiotic drug) is used for the treatment of many diseases such as tuberculosis, skin diseases, etc.

Iron(II) plays a very important role in biological systems. It is an established fact that the oxygen transport *in-vivo* takes place through the iron complex i.e.hemoglobin. Several other important roles of irons are also well known.

The biochemical, pharmacological and medicinal importance of metal-drug complexes is very well established /1,4/. In continuation to the work done in our laboratory on the studies of electrochemical, bioinorganic, antibacterial and pharmacological behavior of some metal-drug complexes /5,6/ the present paper discusses the role of biometal ion Fe(II) in the physicochemical and bioinorganic behavior of SFX and deals with the said studies on the Fe(II)-SFX complex.

### EXPERIMENTAL

### Instrumentation

All the polarograms/voltammograms were recorded on an Elico (India) DC polarograph, model 357 and an Elico (India) pulse polarograph, model CL-90, respectively, coupled with an x-y polarocard model LR-101 and LR-101P. The polarographic cell consists of an electrode assembly having a dropping mercury electrode (working electode), a saturated calomel electrode (reference electrode), and a coiled platinum wire (auxiliary electrode). A Systronics digital pH meter-335 was used for the pH measurements. The IR spectrum of solid complex was recorded using KBr pellets on an IR spectrophotometer – Shimadzu, Japan, Model 470.

### Chemicals and Reagents

The chemicals used were of Analar/BDH grade. The SFX ($C_{19}H_{22}F_2N_4O_3$) sample was supplied to us as a free gift by Ranbaxy Laboratories Gurgaon, India. Doubly distilled water and absolute ethanol (55:45v/v) was used as solvent. pH adjustments were made using dilute solutions of HCl, NaOH whenever necessary.

### Electrochemical Behavior SFX

Qualitative and quantitative studies on SFX were carried on by DCP /7/ and DPP method. Experimental sets were prepared by keeping overall ammonium tartrate concentration fixed at 0.1M. The SFX concentration was varied from 0.0 mM to 1.0 mM. The pH of the test solutions was adjusted to 6.0. The solution was deaerated with purified $H_2$ gas. Polarograms were recorded keeping the initial potential set to -1.3 volts.

The analysis of SFX in pharmaceutical formulation i.e. sparx-100 (tablet) was performed by dissolving the tablet in 100 ml of water alcohol (45:55 v/v) and following the earlier discussed polarographic and amperometric procedures.

### Preparation of Complex

For the study of stoichiometry and formation constant of the complex, Lingane's polarographic method was used. Experimental sets were prepared by keeping overall iron (metal ion) and potassium chloride concentration fixed at 1mM and 0.1M respectively. The ligand concentration was varied from 0.0 to 15 mM. The pH of the test solution was adjusted to 5.0. The solution was deaerated with purified $H_2$ gas. Polarograms were recorded keeping the initial potential set to -1.1 volts.

The amperometric titrations were performed on a manually operated set-up equipped with a polyflex galvanometer (sensitivity $8.1*10^{-9}$) and an Ajco varnier potentiometer. DME was used as an indicator electrode and a calomel electrode served as reference electrode. The capillary characteristic of the DME had a $m^{2/3}$, $t^{1/6}$ value of 2.13 $mg^{2/3}$ $sec^{-1/2}$ at 60cm effective height of mercury column. pH measurements were made on a Systronics pH meter model 335 using a glass and a calomel electrode. All the measurements were made at 25° C.

The experimental sets were prepared by taking different but known amounts of Fe(II) in the cell to which an appropriate amount of KCl (supporting electrolyte ) was added to make its overall concentration 0.1M. The pH was then adjusted to 5.0; it was then titrated against the standard solution of SFX (pH 5.0) at –1.6 V vs saturated calomel electrode (the plateau potential of Fe(II)). After each addition of the titrant the current was read on the galvanometer and the current versus volume of titrant added was plotted.

### Synthesis of the Solid Complex

A brown coloured solid complex was synthesized by refluxing the 1:1 aqueous solution of ferrous ammonium sulphate and SFX solution in water and ethyl alcohol (55:45v/v) for about 5 hr. The complexation was marked by precipitation after reducing the volume of reaction mixture to one fourth of the original volume .The product was filtered, washed, dried over $P_4O_{10}$ and stored.

The results of elemental (C, H, N and O) analysis on the drug and its Fe(II)-SFX were furnished by the CDRI, Lucknow, India. The complexometric method was used for the estimation of iron in the complex /8/.

### Biological Study of Fe(II) SFX Complex

Biochemical applications are in demand nowadays. The activity of bacteria on several compounds gives more important information about the complex. So it prompted us to screen the complex and precursors to find out which part of the molecule is actually responsible for its physiological activity.

### Antibacterial Studies

Raper's paper disc method /9/ was followed for the antibacterial screening of Fe(II)-SFX complex against some pathogenic bacteria, viz. *Staphylococcus aureus, Salmonella typhi, Basillus subtalis* and *Escheria coli.* The percentage inhibition was calculated using the following formula /9/.

$$\% \text{ Inhibition} = \frac{a-b}{a} * 100$$

where 'a' represents the diameter of zone of inhibition for control and 'b' for the complex.

### RESULTS AND DISCUSSION

SFX produced a well defined polarographic wave/peak in 0.1M ammonium tartrate pH 6.0±0.1 with $E_{½}$ =-1.45, $E_p$=-1.49 V vs SCE. Its wave height was found to be proportional to the concentration of SFX (Fig 1 and 2). The polarographic analysis of SFX in the pharmaceutical formulation sparx-100, tablet revealed 100mg SFX per tablet, which is in good agreement with that claimed by the manufacturer.

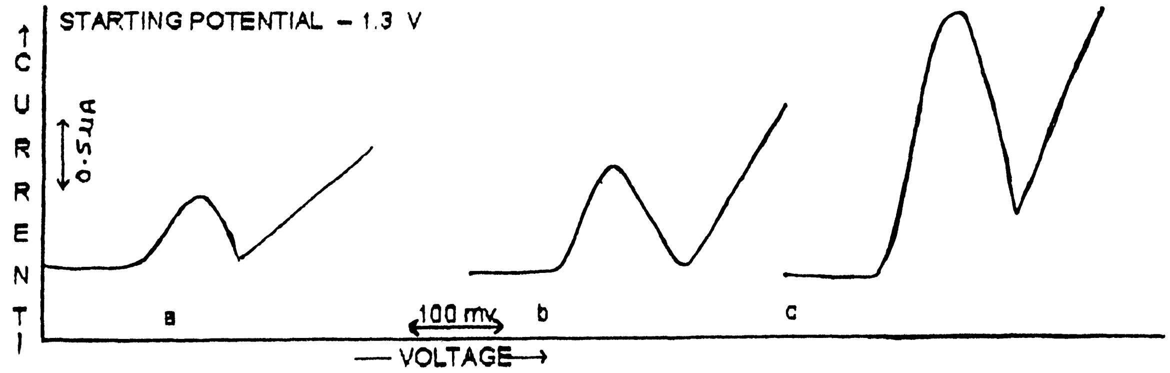

**Fig. 1:** Differential pulse polarogram of Sparfloxacin in 0.1 M ammonium tartrate pH 6.0 ± 0.1. a. 0.125 mM, b. 0.250 mM, c. 0.50 mM.

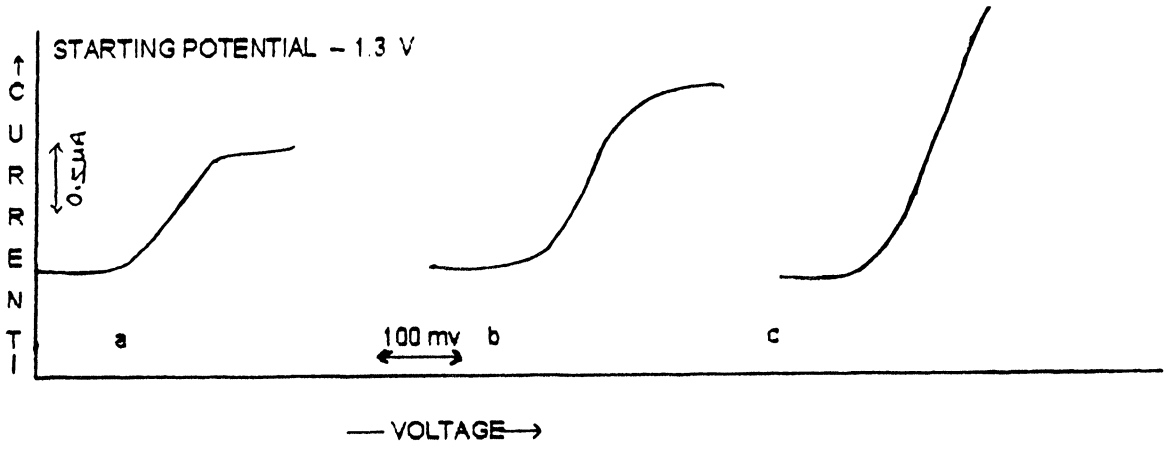

**Fig. 2:** Direct current polarogram of Sparfloxacin in 0.1 M ammonium tartrate pH 6.0 ± 0.1. a. 0.125 mM, b. 0.250 mM, c. 0.50 mM.

### Polarographic Study of M: L Complexation Equilibrium

Both Fe(II) and its complex with SFX ligand produce a reversible two- electron reduction wave in 0.1M KCl at pH 5.0. The number of electrons involved in the electrode reaction was confirmed by the plots of log (i /$i_d$-i) vs E. The plot of $i_d$ vs $\sqrt{h}_{corr}$. proved the reduction to be diffusion-controlled.

The complex formation between Fe(II) and SFX (Fig. 3) was revealed by the shift in half-wave potential of Fe(II) metal ion to a more electronegative value and decrease in the height of the diffusion current with gradual increase of the SFX concentration .

Plots of $\Delta E_{1/2}$ (shift in the $E_{1/2}$) = $(E_{1/2})_c - (E_{1/2})_s$ against log $C_x$ (logarithm of the concentration of the ligand) resulted in a linear plot. The linear plot showed the formation of single complex species in solution. Lingane /11/ treatment of the observed polarographic data revealed 1:1Fe(II)-SFX complex formation with formation constant log $\beta_1$-3.9 (Fig. 4).

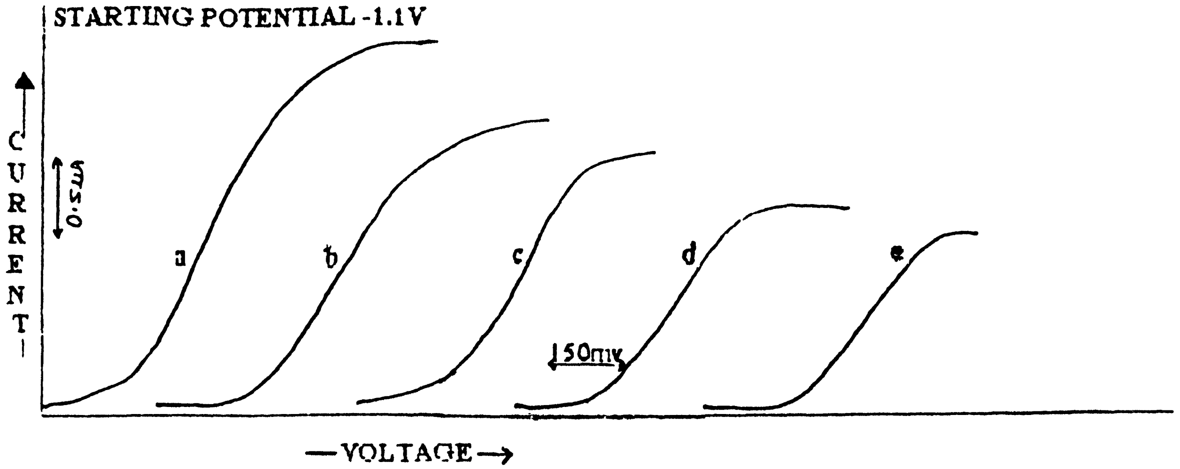

**Fig. 3:** Direct current polarogram of Fe(II) in 0.1 M KCl, pH 5.0 ± 0.1 presence of Sparfloxacin. a. 0.0 mM, b. 0.125 mM, c. 0.250 mM, d. 0.50 mM, e. 0.75 mM.

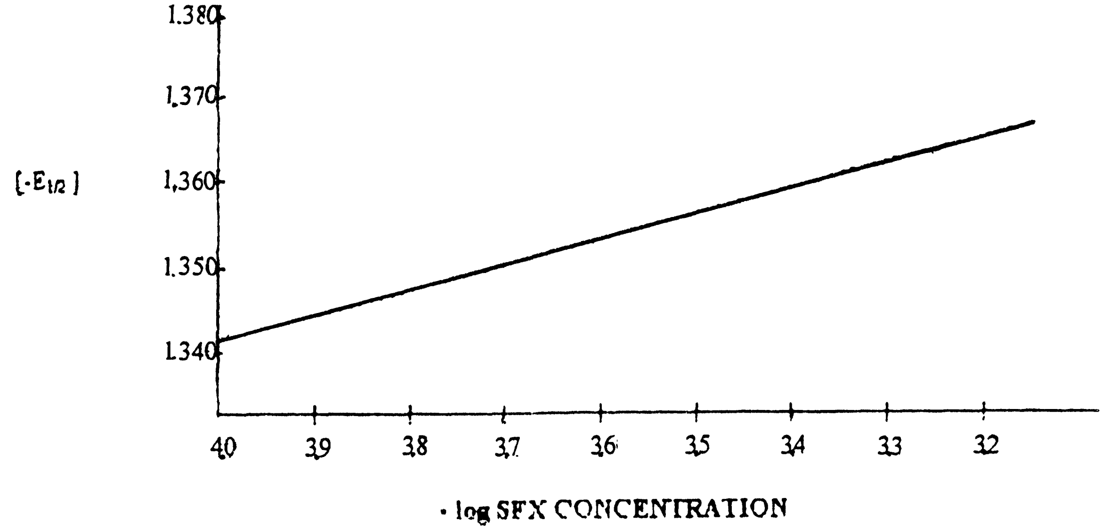

**Fig. 4:** Plot of $-E_{1/2}$ vs log Cx for Fe(II)-Sparfloxacin complex.

### Amperometric Determination of SFX with Fe(II)

Fe(II) gives a well defined polarographic wave in 0.1M KCl at pH 5.0 ± 0.1. The diffusion current was found proportional to its concentration. The SFX does not produce any wave under the said experimental condition. The plateau potential for the polarographic wave of Fe(II), – 1.6 V vs Hg pool anode was applied for carrying out amperometric titration. Fe (II) was taken as titrate, and the drug solution was taken as titrant. The current volume plots resulted in an L shaped curve (Fig. 5). The end point as located by a graphic method revealed a metal to drug ratio of 1:1, which is in agreement with the author's observation on the metal:ligand complexation equilibrium using a polarographic method.

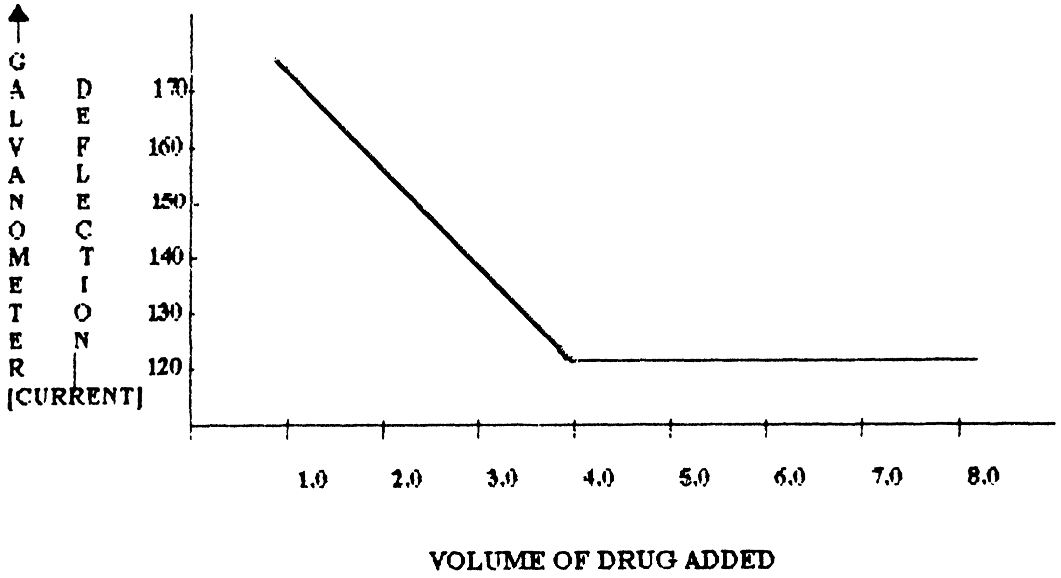

**Fig. 5:** Amperometric titration of 10 mM / 50 ml analyte Fe(II) with 2.5 mM SFX in 0.1 M potassium chloride, pH 5.0 ± 0.1.

### Elemental Analysis

The results of elemental analysis on the drug and its complex with Fe(II) are depicted in Table 1. The table revealed a 1:1 metal:drug ratio in the complex, which supports the author's finding using polarographic and amperometric methods.

### IR-Spectra

Some important frequencies of IR signals of SFX and its Fe(II) complex are listed in Table 2. The stretching vibration due to (C=O) and (N-H) in the IR spectrum of the drug appear as a band at 1710 $cm^{-1}$ and a doublet between 3300-3450 $cm^{-1}$ /12,13/ and are shifted as a band at 1700 $cm^{-1}$ and as a broad and reduced band at 3150 $cm^{-1}$ respectively, in the spectrum of Fe(II)-SFX complex. Thus, it could be concluded that oxygen of carboxylic acid and nitrogen of N-H group are involved in complex formation with the metal ion.

### Antibacterial Studies

Antibacterial activity of the compound (0.5M Conc.) is depicted in Table 3 showing the various bacteria. This complex was found to be more toxic against bacteria. In the case of *Basillus subtalis, Salmonella typhi, Escheria coli* and *Staphylococcus aureus,* the percentage inhibition due to Fe(II)-SFX complex is -18.0, -16.6, -15.7 and -13.6 respectively over the parent drug control.

**Table 1**

Analytical Data of SFX and Fe(II) SFX Complex % Calculated/Found

| Compound | Metal | C | H | N | O | F |
|---|---|---|---|---|---|---|
| SFX | -<br>- | 58.1<br>(58.5) | 5.65<br>(5.68) | 9.69<br>(9.72) | 12.23<br>(12.27) | 14.28<br>(14.35) |
| Fe(II)-SFX complex | 12.74<br>(12.71) | 51.16<br>(51.19) | 4.9<br>(4.93) | 8.0<br>(7.8) | 10.7<br>(10.9) | 12.5<br>(12.9) |

**Table 2**

Principal IR Frequencies ($cm^{-1}$) of SFX and Fe(II)-SFX Complex

| SFX | Assignment | FE(II)-SFX |
|---|---|---|
| 920 | N-H wagging vibration | 920 |
| 1025 | Region of C-F absorption | 1025 |
| 1290 | C=O stretching aromatic conjugation | 1290 |
| 1435 | C-N ring stretching | 1435 |
| 1630 | C=C stretching in ring six member vibration | 1630 |
| 1700 | C=O stretching cyclic (COOH) | 1720 |
| 3450 | Symmetrical N-H stretching | 3150 |

**Table 3**

Antibacterial Activity of Fe(II)-SFX Complex

| **Micro-Organism Bacteria** | **Zone of Inhibition** | | **% Inhibition** |
|---|---|---|---|
| | **Drug(a)*** | **Complex(b)**** | |
| Staphylococcus aureus | 14 | 17 | -21.4 |
| Salmonell typhi | 18 | 23 | -27.7 |
| Escheria coli | 12 | 14 | -16.6 |
| Basillus subtitis | 16 | 19 | -18.75 |

Including diameter of filter paper disc 6 mm

* Drug

** Drug-metal complex mg/ml (equivalent 1 mg/ml)

### CONCLUSION

The polarographic/amperometric methods could be successfully used for the qualitative and quantitative analysis of SFX in pharmaceutical formulation, i.e. sparx-100, and could be recommended for its use for quality control purposes in the drug industry. In addition, in view of the increased bacterial potency it may be recommended to the therapeutic experts for its possible use as a more potent antibiotic drug.

### ACKNOWLEDGEMENT

The authors express their sincere thanks to Prof. S.P. Banerjee, Head, Department of Chemistry, Dr. Harisingh Gour University, Sagar (M.P.), India for providing laboratory facilities.

### REFERENCES

1. L. Wang, Z. An, K. Jia, X. Zhang and H. Huang, *J. Indian Chem. Soc.*, **78**, 305 (2001).
2. S. Narad, N.N. Mishra, P. Pandey, A. Kumar and K.S. Pitre, *Analyt. Letters,* **28**(11), 2005(1995).
3. R. Qureshi and S.A. Iqbal, *Oriental J. Chem,* **15**(1), 44(1999).
4. A.V. Trivedi, J. Shukla and K.S. Pitre, *Ind. J. Exp. Biol.,* **36,** 1125 (1998).
5. R. Das and K. S. Pitre, *J. Indian Chem. Soc.,* **78**, 257 (2001).
6. J. Shukla and K.S. Pitre, *J. of Physiol. and Pharmacol.,* **42**(2), 223 (1998).
7. N.Y. Sreedhar, K. Samatha and B.R. Ravin, *ISA*, **36**(7), [[page numbers]] (2000).
8. J. Bassett, R.C. Denney, G.H. Jeffery and J. Mendham, *Vogel's Textbook of Quantitative Analysis* $4^{th}$ ed., Longman, London, 1978; p.324.
9. K.B Raper, D.F. Alexander and R.D. Coghill, *J. Bacterial,* **48,** 693(1994).
10. R. Kumar, G. Giri and Niza Muddin, *J. Indian Chem Soc.*, **64**, 571 (1988).
11. J.J. Lingane, *Chem Rev*, **1**, 29 (1941).
12. J.R. Dyer, *Applications of Absorption Spectroscopy of Organic Compounds,* Prentice-Hall of India Pvt. Ltd., New Delhi, 1974; p. 35.
13. K. Nakamoto, *IR and Raman Spectra of Inorganic and Co-ordination Compounds,* $3^{rd}$ ed. Wiley Interscience, New York, 1977.

